# Tératome cancérisé: à propos d’un cas avec revue de la littérature

**DOI:** 10.11604/pamj.2017.27.61.11567

**Published:** 2017-05-29

**Authors:** Abdi Ahmed Bonahy, Houssam Sabbah, Ahmed Haiba Med Vadell, Nacer Eddine Med Baba

**Affiliations:** 1 Département Mère et Enfant, Faculté de Médicine de Nouakchott, Mauritanie; 2 Maternité du Centre Hospitalier National (CHN) de Nouakchott, Mauritanie; 3 Service d’Anatomopathologie du Centre Hospitalier National (CHN) de Nouakchott, Mauritanie

**Keywords:** Carcinome épidermoïd, kyste dermoïde ovarien, tératome kystique mature, transformation maligne, Squamous cell carcinoma, ovarian dermoid cyst, mature cystic teratoma, malignant transformation

## Abstract

Parmi les tumeurs germinales de l’ovaire, on retrouve les kystes dermoïde dans 10 à 20% des cas. Dans 1 à 2 % des cas, une transformation maligne en kyste dermoïde cancérisé a été décrite(KDC). Le traitement, est un véritable sujet à controverse, Chez la femme en âge de procréer et pour les stades débutants, une annexectomie unilatérale sans traitement adjuvant est préconisée. Quant aux cas où il s’agit d’une femme ménopausée, certaines équipes réalisent une chirurgie élargie et ceci quelques soit le stade. Nous rapportons le cas KDC chez une patiente ménopausée traitée chirurgicalement et dont l’évolution a été favorable.

## Abstract (en)

Dermoid cysts account for 10-20% of ovarian germ cell tumors. Malignant transformation within ovarian dermoid cyst has been reported in 1-2 per cent of the cases. The treatment remains controversial. In women of childbearing age and in the first stages of the disease unilateral annexectomy without adjuvant therapy is recommended. In menopausal women extensive surgery is sometimes performed, no matter what stage of the disease the person is experiencing. We report the case of a menopausal patient with malignant transformation within ovarian dermoid cyst who underwent surgery. She had a favorable outcome.

## Introduction

Les kystes de l’ovaire sont fréquemment observés dans la pathologie gynécologique. S’ils peuvent se présentés sous différents types anatomique et histologique, le véritable problème réside dans le diagnostic et la prise en charge des formes cancérisées. Nous nous intéressons dans ce travail à une forme très rare appelée kyste dermoïde cancérisé Cette forme représente environ 1 à 2% des tératomes kystiques matures (TKM) [1,2]. Dans 80% des cas, il s’agit d’un carcinome épidermoïde [1,3,4]. Nous rapportons le cas KDC chez une patiente ménopausée depuis 10 ans traitée chirurgicalement et dont l’évolution a été favorable.

## Patient et observation

**Figure 1:**
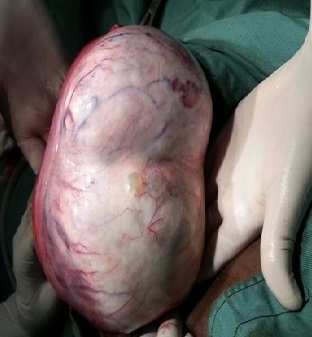
Gros kyste ovarien droit en peroperatoire

Patiente H. N., âgée de 59 ans, consulte dans un tableau de douleurs pelviennes continue à type de pesanteur avec augmentation du volume de l’abdomen évoluant depuis un mois. Il s’agissait d’une grande multipare, ménopausée depuis 10 ans, sans antécédents médico-chirurgicaux. L´examen clinique trouve une masse abdominopelvienne à convexité supérieure sensible. L’examen au spéculum était normal, quant au toucher vaginal, il a permis de mettre en évidence une masse dure, mobile et séparée de l’utérus par un sillon de séparation. L’échographie a permis la mise en évidence d’une masse d’échostructure mixte solidokystique de 20 cm de grand axe évoquant un tératome. Un acte chirurgical par la voie classique est alors décidé et une laparotomie médiane a été réalisée. Elle a mis en évidence une tumeur ovarienne droite bien limitée adhérente à l’appendice Figure 1.

**Figure 2:**
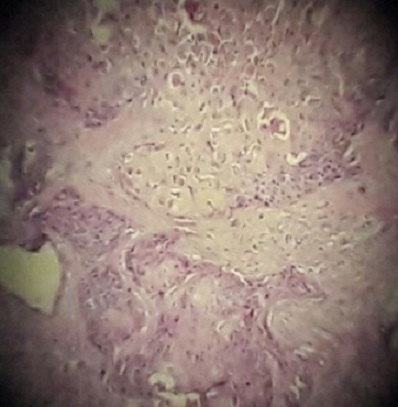
Aspect anatomopathologique du carcinome épidermoïde mature bien différencié sur tératome mature

**Figure 3:**
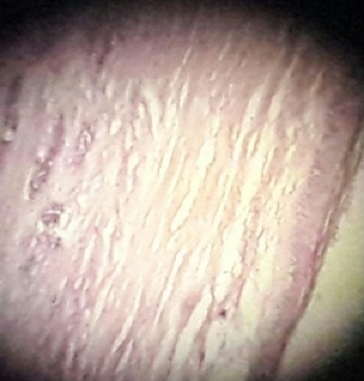
Gros kyste ovarien droit en peroperatoire

Devant l’impossibilité de réaliser un examen extemporané, une hystérectomie totale avec annexectomie bilatérale est alors réalisée. Les suites opératoires étaient simples. Les résultats de l’examen anatomopathologiquee: carcinome épidermoïde bien différencié, infiltrant et mature, sur un tératome mature de l’ovaire stade pT1c (Figure 2, Figure 3). Nous avons discuté avec le centre national d’oncologie l’intérêt d’un traitement adjuvant, la décision était de surveiller la patiente sans traitement. Depuis, un contrôle bi annuel est réalisé et aucune récidive n’a été observée.

## Discussion

Parmi les tumeurs germinales de l’ovaire, on retrouve les kystes dermoïde dans 10 à 20% des cas [1,2]. Dans 1 à 2 % des cas, une transformation maligne de cette pathologie survient [1,2]. L’âge de survenue dece cancer chez notre patiente a été de 58ans ce qui correspond à l’âge moyen de survenue rapporté par plusieurs auteurs et qui est de 54 ans [5–7]. Moins observé en périodepériménopausique, il est fréquent chez la femme ménopausée depuis quelques années. Notre patiente était ménopausée depuis 10ans. Le symptôme le plus souvent observé est la douleur pelvienne basse à type de pesanteur [8]. Quant à la constipation, elle est la résultante des phénomènes de compression.

Si l’échographie constitue l’examen radiologique de choix dans le diagnostic et la surveillance des tératomes, elle ne permet en aucun cas de déceler des signes de transformation maligne [9]. Dans le cas où la Tomodensitométrie abdominopelvienne est réalisée, elle confirme la présence d’un processus abdominopelvien de densité liquidienne, avec des calcifications arrondies et une paroi épaissie irrégulière en faveur d’un tératome ovarien. Dans notre cas, nous nous étions basé sur les résultats de l’échographie pour évoquer le diagnostic du tératome ovarien. Les signes de cancérisation probables n’avaient pas été recherchés.

Quelques auteurs se sont attardés à rechercher des signes de malignités. Bien que rares, certains éléments cliniques, biologique et radiologiques seraient, selon ces auteurs, évocateurs de la possible malignité. Ils rapportent que l’âge supérieur à 40 ans est l’un de ces critères avec un pic d’incidence de cancérisation situé entre 45 et 60 ans [2]. La taille supérieure à 99 mm, l’augmentation de la taille d’un kyste dermoïde en période ménopausique ou toute croissance supérieure à deux centimètres par an en période d’activité génitale doivent aussi faire suspecter la transformation maligne d’un kyste dermoïde [5]. Parmi les éléments biologiques étudiés, Seule l’analyse du squamous cell carcinoma (SCC) pourrait apporter un indice quant à la transformation maligne du kyste dermoïde. Cependant, un faible taux de ce marqueur ne permet pas d’éliminer la cancérisation [8]. Sur le plan radiologique, si l’échographie seule ne permet pas d’évoquer la malignité, couplée au doppler, elle permettrait de révéler la présence d’un contingent malin par l’association d’un flux sanguin intratumoral, d’un index de pulsabilité ainsi qu’une résistance moyenne abaissé [10]. Par ailleurs, la TDM ainsi que l’IRM évoquerait la présence d’un contingent malin comme étant une croissance invasive à bords irréguliers franchissant la paroi du kyste avec un rehaussement marqué après l’ injection du produit de contraste et par la présence d’éléments solides à l’intérieur d’un contenu à majorité liquidien [3].

Quant au traitement, il s’agit d’un véritable sujet à controverse. Chez la femme en âge de procréer et pour les stades débutants, une annexectomie unilatérale sans traitement adjuvant est préconisée. Quant aux cas où il s’agit d’une femme ménopausée, certaines équipes réalisent une chirurgie élargie et ceci quelques soit le stade [10,11]. Le rôle de la chimiothérapie n’est pas encore codifié. Devant l’efficacité des agents alkylants de façon générale sur les cancers de l’ovaire, certaines équipes préconisent leur utilisation [11]. Dans la littérature, la radiothérapie, non seulement n’apporte aucun bénéfice, mais en plus apporte son lot de complications [3,11].

Dans notre cas, et après un travail conjoint avec le centre national de cancérologie, nous avons décidé d’une surveillance clinique et radiologique tout les 6mois. Avec un recul de 3ans, aucune récidive n’a été observée.

## Conclusion

Les kystes dermoïde constituent une entité rare dans la pathologie gynécologique. Une forme plus rare peut toucher la femme surtout ménopausée, il s’agit du tératome cancérisé. La confirmation du diagnostic reste l’anatomo-pathologie même si la tomodensitométrie apporterait quelques signes évocateurs. Quant au traitement, grand sujet de discussion, il nécessite une codification pour permettre une prise en charge adapté de cette pathologie.

## Conflits d’intérêts

Les auteurs ne déclarent aucun conflit d'interêts.
